# Genetic Background Studies of Eight Common Beta Thalassemia Mutations in Thailand Using β-Globin Gene Haplotype and Phylogenetic Analysis

**DOI:** 10.3390/genes13081384

**Published:** 2022-08-03

**Authors:** Rossarin Karnpean, Wanicha Tepakhan, Prame Suankul, Sitthikorn Thingphom, Apichaya Poonsawat, Naritthakarn Thanunchaikunlanun, Rotsakorn Ruangsanngamsiri, Wittaya Jomoui

**Affiliations:** 1 Maha Chakri Sirindhorn Medical Center, Department of Pathology, Faculty of Medicine, Ongkharak Campus, Srinakharinwirot University, Nakhon Nayok 26120, Thailand; rossarink@g.swu.ac.th; 2 Department of Pathology, Faculty of Medicine, Prince of Songkla University, Songkhla 90110, Thailand; wanicha.te@psu.ac.th; 3 Faculty of Medicine, Srinakharinwirot University, Nakhon Nayok 26120, Thailand; prame.suankul@g.swu.ac.th (P.S.); sitthikorn.thingphom@g.swu.ac.th (S.T.); apichaya.psw@g.swu.ac.th (A.P.); naritthakarn.natt@g.swu.ac.th (N.T.); rotsakorn.khem@g.swu.ac.th (R.R.)

**Keywords:** haplotype, linkage analysis, phylogenetic tree, thalassemia, Thailand

## Abstract

Single nucleotide polymorphisms are informative for haplotype analysis associated with genetic background and clinical linkage studies of β-thalassemia mutations. Hence, the aim of this study was to investigate five polymorphisms (codon 2 (C/T), IVS II-16 (C/G), IVS II-74 (G/T), IVS II-81 (C/T) and the *Hinf* I (T/A) polymorphism) on the β-globin gene, related to eight common β-thalassemia mutations in Thailand, including NT-28 (A > G), codon 17 (A > T), codon 19 (A > G), HbE (G > A), IVS I-1 (G > C), IVS I-5 (G > C), codon 41/42 (-TTCT) and IVS II-654 (C > T). The strongest LD (100%) between the β-thalassemia mutation allele and all five SNPs was found in NT-28 (A > G), codon 17 (A > T) and codon 19 (A > G). In the haplotype analysis, we found three haplotypes (H1, H2 and H7) related to Hb E, whereas we only found two haplotypes related to codon 41/42 (-TTCT) (H1, H3) and IVS I-1 (G > C) (H3, H4). Of interest is the finding relating to a single haplotype in the remaining β-thalassemia mutations. Furthermore, phylogenetic tree analysis revealed three clusters of these common β-thalassemia mutations in the Thai population and enabled us to determine the origin of these mutations. Here, we present the results of our study, including four intragenic polymorphisms and the finding that the *Hinf* I polymorphism could be informative in genetic background analysis, population studies and for predicting the severity of β-thalassemia in Thailand.

## 1. Introduction

β-Thalassemia mutation is a β-globin gene defect on chromosome 11. More than 200 mutations have been reported worldwide (http://globin.cse.psu.edu; accessed on 20 June 2022). These genetic defects result from single nucleotide substitutions and deletions or insertions of oligonucleotides that lead to a shift in the reading frame. Severe β-thalassemia diseases are related to homozygous β-thalassemia, compound heterozygous β-thalassemia and Hb E in Southeast Asia and Thailand [1,2]. In Thailand, more than 30 β-thalassemia mutations have been reported. There is a high frequency of the β-thalassemia carrier in each region of Thailand (between 3% and 9%). However, it has been reported that eight common β-thalassemia mutations in Thailand (including codon 41/42 (-TTCT), codon 17 (A > T), NT-28 (A > G), IVS II-654 (C > T), codon 19 (A > G), codon 26; HbE (G > A), IVS I-1 (G > C) and IVS I-5 (G > C)) represent more than 85% of the total β-thalassemia mutations [3,4,5]. Several previous studies have reported the genetic relationship between populations, chromosome background of the gene and linkage analysis, as well as differences in clinical phenotypic expression using haplotype and phylogenetic analyses [6,7,8]. Single nucleotide polymorphisms (SNPs) within the β-globin gene cluster on chromosome 11 have been found, with seven–eight polymorphic sites commonly studied [9,10]. However, because of the presence of recombination “hot spots” in the region, the β-globin gene haplotype can be divided into 5′ and 3′ sub-haplotypes, corresponding to regions 5′ and 3′ of the “hot spot”. These 5′ haplotypes are limited to study of the genetic background of β-thalassemia mutations. Only two DNA polymorphisms on 3′ haplotypes are useful for studying the genetic background of β-globin genes: *Ava* II (IVS II-16) (rs10768683; C > G), located on the β-globin gene, and *Hinf* I (rs10837631; T > A), located on the 3′ β-globin gene [11,12]. Newly intragenic polymorphisms of the β-globin gene are informative polymorphisms in haplotype analysis associated with genetic background and clinical linkage studies of β-thalassemia mutations [13,14,15]. However, studies of these intergenic polymorphisms have yet to be comprehensively reported for the Thai population [16]. Hence, the aim of this study was to construct four intragenic polymorphisms (codon 2 (C/T), IVS II-16 (C/G), IVS II-74 (G/T) and IVS II-81 (C/T)) and one 3′ haplotype (*Hinf* I (T/A)) on the β-globin gene, related to eight common β-thalassemia mutations in Thailand, using haplotype and phylogenetic analysis.

## 2. Materials and Methods

### 2.1. Samples

Ethical approval for the study was obtained from the Institutional Review Board of Srinakharinwirot University, Thailand (SWUEC/E/M-029/2565). Archival DNA specimens were obtained from the Department of Pathology, Maha Chakri Sirindhorn Medical Center, Faculty of Medicine, Srinakharinwirot University (SWU), Nakhon Nayok, Thailand. In total, 163 leftover DNA samples with eight common β-thalassemia mutations were recruited for the study (codon 41/42 (-TTCT), codon 17 (A > T), NT-28 (A > G), IVS II-654 (C > T), codon 19 (A > G), HbE (G > A), IVS I-1 (G > C) and IVS I-5 (G > C)) and the wild type. The 163 samples included 23 compound heterozygous β-thalassemia/Hb E, 31 homozygous Hb E and 85 heterozygous β-thalassemia; 24 wild types were also studied. The hematological parameters were performed on hematology automation (Sysmex XN3000, Sysmex, Kobe, Japan). Hb typing analysis was conducted using capillary electrophoresis (Capillarys 2; Sebia, Lisses, France). Identification of common β-thalassemia mutations in Thailand (including codon 41/42 (-TTCT), codon 17 (A > T), NT-28 (A > G), IVS II-654 (C > T), codon 19 (A > G), codon 26; HbE (G > A), IVS I-1 (G > C) and IVS I-5 (G > C)) was performed routinely in our laboratory using polymerase chain reaction (PCR) methods, as described elsewhere [2,3].

### 2.2. Genotyping of Intragenic β-Globin Gene Polymorphisms and the Hinf I Polymorphism

Four intragenic β-globin gene polymorphisms, including codon 2 (C/T), IVS II-16 (C/G), IVS II-74 (G/T) and IVS II-81 (C/T), were genotyped using direct DNA sequencing on an ABI PRISM™ 3130 XLanalyzer (Applied Biosystems, Foster City, CA, USA), as described elsewhere [5]. Only samples with the codon 41/42 (-TTCT) mutation (four polymorphisms) were genotyped, with multiplexing targeted sequencing, using a barcode and a next-generation sequencing platform. *Hinf* I polymorphism genotyping was conducted using allele-specific PCR, as described elsewhere [17]. The basic statistics for five polymorphisms were calculated, including the allele frequency, genotype frequency and minor allele frequency (*p*-value < 0.05), using PEAS V1.0: a package for elementary analysis of SNP data. The Fisher’s exact test revealed the derived allele frequency (DAF), and MINITAB release 14.12.0 statistical software was used for the calculations, indicating a significant difference between the wild type and β-thalassemia genotypes in biological polymorphic markers. A *p*-value < 0.05 was considered to be statistically significant.

### 2.3. Haplotype and Phylogenetic Analysis

Haplotype patterns (constructed using data from the four intragenic β-globin gene polymorphisms and *Hinf* I polymorphism, and the pairwise linkage disequilibrium (LD) test) were determined using HAPLOVIEW 4.2 software, which examined haplotypes >0.5%. The Hardy–Weinberg equilibrium (HWE) was tested in β-thalassemia subgroup mutation analysis. The D′ value of the linkage disequilibrium test was calculated and shown in an LD pattern [18]. The phylogenetic tree analysis was constructed with a dendrogram based on the haplotype pattern. DendroUPGMA software (http://genomes.urv.cat/UPGMA/, accessed on 10 May 2022) was used and the Jaccard (Tanimoto) coefficient was applied, with default settings; 100 bootstrap replications were performed. FigTree v.1.4.0 software was then used to design a graphical viewer for the phylogenetic tree.

## 3. Results

The hematological data for 163 subjects are shown in Table 1. The recruited samples were categorized into 14 groups as β-thalassemia genotypes. The most severe form of anemia was found in compound heterozygous β-thalassemia/Hb E cases, represented by low Hb and Hct values, whereas the homozygous Hb E, and β-thalassemia carrier showed minimal anemia with hypochromic (MCH), microcytic RBC (MCV). Hb analyses were also included and related to β-thalassemia genotypes.

Four intragenic β-globin gene polymorphisms (codon 2 (C/T), IVS II-16 (C/G), IVS II-74 (G/T) and IVS II-81 (C/T)) located on the β-globin gene and the *Hinf* I polymorphism on the 3′ β-globin gene were genotyped using DNA sequencing and AS-PCR, respectively. Table 2 shows the allele frequency (ancestral allele and derived allele), categorized into 11 groups. Compound heterozygous β-thalassemia/Hb E accounted for one group because this was limited in the total samples. The derived allele frequency (DAF) of each polymorphism (T of codon 2, G of IVS II-16, T of IVS II-74, T of IVS II-81 and T of the *Hinf* I polymorphism) was calculated and compared with the β-thalassemia genotypes and wild type. Fisher’s exact test revealed a significant DAF difference between the wild type and five β-thalassemia genotypes (β^E^/β^thal^, β^E^/β^E^, β^N^/β^17^, β^N^/β^19^ and β^N^/β^IVS II-654^) in codon 2. Furthermore, the DAF showed a significant difference between the wild type and four β-thalassemia genotypes (β^E^/β^E^, β^N^/β^17^, β^N^/β^19^ and β^N^/β^IVS II-654^) in IVS II-16, whereas the DAF in IVS II-74 showed a significant difference between the wild type and three β-thalassemia genotypes (β^N^/β^41/42^, β^N^/β^IVS I-1^ and β^N^/β^IVS II-654^). In the case of the *Hinf* I polymorphism, the DAF showed a significant difference between the wild type and five β-thalassemia genotypes (β^E^/β^E^, β^N^/β^41/42^, β^N^/β^17^, β^N^/β^19^ and β^N^/β^IVS I-1^). However, a significant difference was not found between the wild type and β-thalassemia mutation in the case of the IVS II-81 polymorphism.

β-Globin gene haplotypes were constructed using HAPLOVIEW 4.2 software based on five polymorphisms, including four intragenic β-globin gene polymorphisms (codon 2 (C/T), IVS II-16 (C/G), IVS II-74 (G/T) and IVS II-81 (C/T)) and the *Hinf* I polymorphism. The genotype distribution of β-thalassemia mutations and five polymorphisms was analyzed for the Hardy–Weinberg equilibrium (HWE). All β-thalassemia mutations and five polymorphisms were found in the Hardy–Weinberg equilibrium (*p*-value > 0.001), except for the Hb E mutation (codon 26). Eight common β-thalassemia mutations and wild types were found to have seven different haplotypes (H1–7) (Table 3). The wild type was found to have five different haplotypes, the most common of which were haplotype H1 (T-G-T-C-A) (42.11%) and haplotype H2 (C-C-T-C-T) (38.00%). Common β-thalassemia mutations were found to be related to five β-globin gene haplotype patterns, and each mutation had a different haplotype pattern. Codon 26 (Hb E) was related to three haplotypes, including haplotype H1 (T-G-T-C-A) (10.59%), haplotype H2 (C-C-T-C-T) (87.06%) and haplotype H7 (T-G-T-C-T) (2.35%). Codon 41/42 (-TTCT) was found to have two haplotypes: H1 (T-G-T-C-A) (27.78%) and H3 (C-C-G-C-A) (72.22%). The IVS I-1 (G > C) mutation was related to H3 (C-C-G-C-A) (50.00%) and H4 (C-C-T-C-A) (50.00%). The remaining five common β-thalassemia mutations were found to have only one haplotype, indicating a single origin of β-thalassemia mutations in Thailand. Haplotype H1 (T-G-T-C-A) was found to be 100% for codon 17 (A > T), NT-28 (A > G) and IVS I-5 (G > C). Haplotype H2 (C-C-T-C-T) and haplotype H3 (C-C-G-C-A) were found to be 100% for codon 19 (A > G) and IVS II-654 (C > T), respectively.

**Figure 1:**
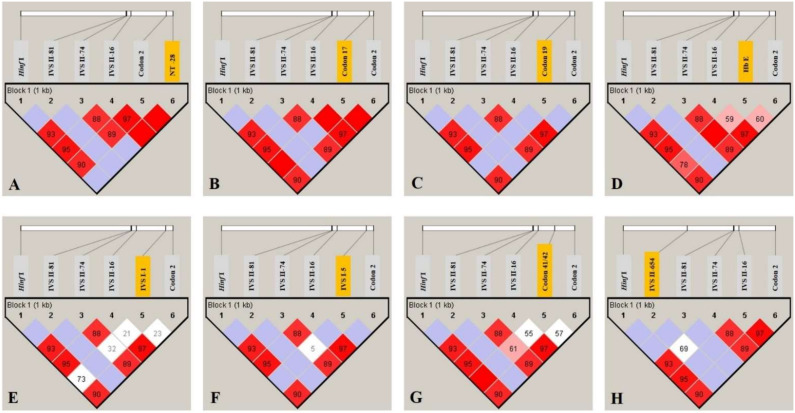
Pairwise analysis of linkage disequilibrium (LD) between five SNPs and each β-thalassemia mutation in a pooled sample of 163 subjects ((**A**–**H**); NT-28 (A > G), codon 17 (A > T), codon 19 (A > G), codon 26; HbE (G > A), IVS I-1 (G > C), IVS I-5 (G > C), codon 41/42 (-TTCT) and IVS II-654 (C > T), respectively). The different color in the haploview plot follows the standard color scheme for haploview: white, |D′| < 1, LOD < 2; shades of pink, |D′| < 1, LOD ≥ 2; blue, |D′| = 1, LOD < 2; red, |D′| = 1, LOD ≥ 2.

The results of pairwise analysis of linkage disequilibrium (LD) between five SNPs and each β-thalassemia mutation in the pool of 163 subjects are shown in Figure 1. A strong LD for each β-thalassemia mutation allele with all five SNPs was found (see Figure 1, with the D′ value) (A-H; NT-28 (A > G), codon 17 (A > T), codon 19 (A > G), codon 26; HbE (G > A), IVS I-1 (G > C), IVS I-5 (G > C), codon 41/42 (-TTCT) and IVS II-654 (C > T), respectively). The strongest LD (D′ value = 1; 100%) between the β-thalassemia mutation allele and all five SNPs was found in NT-28 (A > G), codon 17 (A > T) and codon 19 (A > G), where no D′ value was indicated in the LD pattern. The remaining five β-thalassemia mutations were found to have both strong LDs and weak LDs in each SNP.

**Figure 2:**
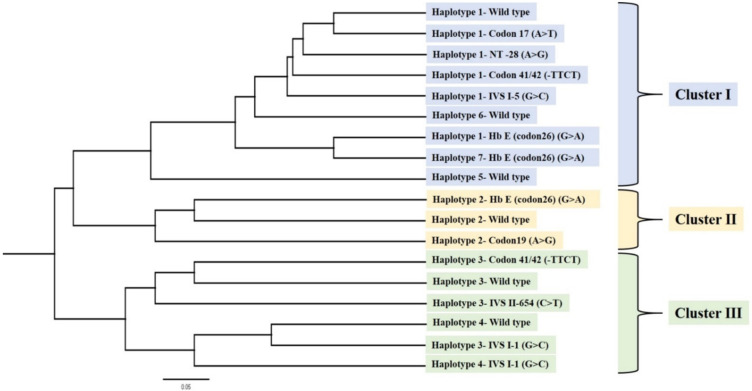
Phylogenetic tree based on haplotype data, constructed using DendroUPGMA software (http://genomes.urv.cat/UPGMA/, accessed on 5 May 2022) with application of the Jaccard (Tanimoto) coefficient, cophenetic correlation coefficient (CP) 0.8148 and the distance matrix based on the Jaccard (Tanimoto) coefficient with 100 bootstrap replicates generated.

Figure 2 shows a phylogenetic tree based on haplotype data, constructed using DendroUPGMA software (http://genomes.urv.cat/UPGMA/, accessed on 5 May 2022) with application of the Jaccard (Tanimoto) coefficient, cophenetic correlation coefficient (CP) 0.8148 and the distance matrix based on the Jaccard (Tanimoto) coefficient; 100 bootstrap replicates were generated. The results represent the evolution of common β-thalassemia mutations in Thailand. Common β-thalassemia mutations and the wild type on the β-globin gene were categorized as three major groups (clusters), as shown in the phylogenetic tree. Cluster I correlated to four β-thalassemia mutations, including NT-28 (A > G), codon 17 (A > T), IVS I-5 (G > C), codon 26; HbE (G > A) and codon 41/42 (-TTCT), whereas Cluster II was related to codon 19 (A > G) and codon 26; HbE (G > A). Cluster III showed three β-thalassemia mutations, including IVS I-1 (G > C), codon 41/42 (-TTCT) and IVS II-654 (C > T). HbE (G > A) (three branches), codon 41/42 (-TTCT) (two branches) and IVS I-1 (G > C) (two branches) were found to have a multi-origin. However, the remaining five β-thalassemia mutations (NT-28 (A > G), codon 17 (A > T), codon 19 (A > G), IVS I-5 (G > C) and IVS II-654 (C > T)) were found to have only a single origin in Thailand, according to the haplotype and phylogenetic tree analysis.

## 4. Discussion

In Thailand, more than 30 β-thalassemia mutations have been reported (ranging from 3% to 9% depending on the region) [2,4,5]. In this study, we focused on eight common β-thalassemia mutations and sought to demonstrate their genetic background in Thailand. Several studies have investigated Hb E and reported the results of haplotype and phylogenetic analysis [19,20,21]. However, haplotype and phylogenetic analyses have yet to be carried out for the intragenic polymorphism within the β-globin gene in Thailand. Here, we report a constructed haplotype of common β-thalassemia mutations based on four intragenic polymorphism β-globin genes and the *Hinf* I polymorphism in Thailand. The novel haplotype constructed in this study may provide a better representation of the actual genetic background of these mutations than those previously constructed. Previous β-globin gene haplotype studies have had the limitation of a recombination hot spot near the 5′ end of the β-globin gene, which may lead to mistakes in detecting mutated alleles [8,11,13,22]. To increase the accuracy of the indirect genetic background analysis, the evaluation of new polymorphic sites and molecular analysis of β-thalassemia are investigated [13].

Table 1 shows that some genotypes of β-thalassemia mutations have only a few samples that may influence the polymorphism analysis, such as the cases of compound heterozygous Hb E/β-thalassemia (codon 19), Hb E/β-thalassemia (IVS I-5), Hb E/β-thalassemia (codon 17). In Table 2 we accumulated these same genotypes as compound heterozygous β-thalassemia/Hb E groups for polymorphisms analysis. Table 2 shows the allele frequency of five polymorphisms within the β-globin gene and the 3′ end of the β-globin gene. In the Thai population, the wild type was found to have high levels of heterogeneity in codon 2 (C/T), IVS II-16 (C/G) and the *Hinf* I polymorphism, with a minor allele frequency (MAF) of more than 30%, whereas IVS II-74 (G/T) and IVS II-81 (C/T) were found to have the least heterogeneity (MAF < 5%). However, two intragenic polymorphisms (IVS II-74 (G/T) and IVS II-81 (C/T)) have been found to have high levels of heterogeneity in other populations [13,14,23]. We compared the β-thalassemia genotype and wild type in each DAF of polymorphisms using the Fisher’s exact test. Compound heterozygous β-thalassemia/Hb E was found to be significantly different for codon 2 (C/T). Homozygous Hb E was found to be different for codon 2 (C/T), IVS II-16 (C/G) and the *Hinf* I polymorphism. Heterozygous β-thalassemia codon 41/42 (-TTCT) and IVS I-1 (G > C) were found to be different for IVS II-74 (G/T) and the *Hinf* I polymorphism, whereas heterozygous β-thalassemia codon 17 (A > T) and codon 19 (A > G) were found to be different for codon 2 (C/T), IVS II-16 (C/G) and the *Hinf* I polymorphism. Lastly, heterozygous β-thalassemia IVS II-654 (C > T) was significant for codon 2 (C/T), IVS II-16 (C/G) and IVS II-74 (G/T). However, compound heterozygous β-thalassemia (NT-28 (A > G))/Hb E, heterozygous β-thalassemia NT-28 (A > G) and heterozygous β-thalassemia IVS I-5 (G > C) were not found to be significantly different from the wild type in all five polymorphisms. Interestingly, several β-thalassemia genotypes were found to be associated with intragenic polymorphisms, which could be a useful marker for linkage analysis and prenatal diagnosis. Furthermore, these intragenic polymorphisms could be routinely investigated in β-thalassemia mutation detection, based on direct DNA sequencing. The strong LD (D′ value = 1) between each β-thalassemia mutation allele (NT-28 (A > G), codon 17 (A > T) and codon 19 (A > G)) with all five SNPs is useful for predicting the genetic background of these β-thalassemia mutations in the Thai population. Furthermore, we found that these β-thalassemia mutations have a single origin, based on their single haplotype pattern.

Haplotypes are information for better understanding of the origin and evolutionary relationships between populations or clinical studies [24,25]. Haplotype analysis revealed five different haplotypes of the wild type in the Thai population, based on intragenic polymorphisms within the β-globin gene and the *Hinf* I polymorphism. Previous studies have reported finding framework 1 (16.32%), framework 2 (44.63%) and framework 3 (39.05%) [19]. In this study we found only framework 1, 2 and 3a, but not framework 3. This may be due to the fact that framework 3 can be differentiated by IVS II-81 (C/T) polymorphisms, whereas the previous study only analyzed 3′ haplotypes, including *Ava* II and *Hinf* I polymorphisms. In this study, we found that the haplotype pattern of Hb E was related to haplotype H1 (framework 3a), H2 (framework 2) and H7 (framework 3a), which is in line with the results of the previous study [19]. The haplotype pattern of other β-thalassemia mutations has yet to be reported. Interestingly, we found two haplotype patterns in codon 41/42 (-TTCT) (H1, H3) and IVS I-1 (G > C) (H3, H4), whereas a single haplotype was found in NT-28 (A > G), codon 17 (A > T), codon 19 (A > G), IVS I-5 (G > C) and IVS II-654 (C > T), indicating that these β-thalassemia mutations have two points of origin and a single origin in the Thai population, respectively.

We constructed a phylogenetic tree of common β-thalassemia mutations in the Thai population, as shown in Figure 2. With regard to the evolution of the β-thalassemia mutations, Cluster I was found to be associated with codon 17 (A > T), NT-28 (A > G), codon 41/42 (-TTCT) and IVS I-5 (G > C), which originate from the same branch, whereas the two Hb E origins were different in the others. This cluster represents the highest level of mutation change (from the root of the tree upwards). Furthermore, the short branch represents younger mutations in this region. Cluster II shows the least amount of evolution and is associated only with codon 19 (A > G) and Hb E, with codon 19 (A > G) also showing a single origin in the Thai population. Cluster III was found to be related to codon 41/42 (-TTCT) and closely to IVS II-654 (C > T), whereas IVS I-1 (G > C) showed two origins that were separate from the two in Cluster III. This phylogenetic tree may provide a better representation than previously reported conceptualizations of the overall genetic background and evolution of common β-thalassemia mutations in Thailand.

## 5. Conclusions

Four intragenic polymorphisms on the β-globin gene and the *Hinf* I polymorphism may be useful markers for linkage analysis with β-thalassemia mutations in Thailand, and all five SNPs are useful for predicting the genetic background of these β-thalassemia mutations. Phylogenetic tree analysis shows demographic details of the evolution of eight common β-thalassemia mutations in Thailand. These polymorphisms should be selected when predicting clinical phenotypic correlations in the future.

## Figures and Tables

**Table 1:** A total of 163 unrelated samples with hematological data (Hb, Hct, MCV, MCH, DCIP), Hb analysis and β-globin gene genotype. The values are indicated as mean with standard deviation or as raw data where appropriate.

Genotypes	*n*	Hb (g/dL)	Hct (%)	MCV (fL)	MCH (pg)	Hb Type	Hb A2/E	Hb F
β^E^/β^41/42^	5	8.2 ± 1.7	24.7 ± 4.7	53.4 ± 4.9	17.7 ± 1.2	EF	58.4 ± 20.3	41.5 ± 20.2
β^E^/β^17^	2	6.8, 5.6	22.9, 20.6	67.4, 78.9	20.0, 21.5	EF	50.0, 62.0	39.0, 38.2
β^E^/β^19^	1	9.8	29.6	54.3	18.0	EA	57.2	4.1
β^E^/β^IVS I-5^	1	5.6	16.5	50.2	17.0	EF	67.4	26.4
β^E^/β^-28^	14	8.8 ± 2.2	26.7 ± 6.3	58.8 ± 6.9	19.2 ± 2.1	EFA, EA	56.6 ± 8.4	16.1 ± 11.1
β^E^/β^E^	31	12.5 ± 1.7	36.5 ± 5.9	60.3 ± 3.2	20.5 ± 1.4	EE	95.8 ± 5.1	3.1 ± 2.2
β^N^/β^41/42^	31	10.3 ± 2.6	32.1 ± 8.3	64.2 ± 4.0	20.9 ± 2.0	A2A	5.5 ± 0.4	2.0 ± 1.6
β^N^/β^17^	23	10.3 ± 2.8	32.5 ± 8.9	58.3 ± 4.2	18.7 ± 2.2	A2A	5.4 ± 0.6	1.7 ± 1.9
β^N^/β^19^	4	11.5 ± 1.1	35.1 ± 3.4	72.1 ± 6.0	23.6 ± 1.5	A2A	4.3 ± 0.3	0.7 ± 0.4
β^N^/β^-28^	9	12.7 ± 2.4	38.9 ± 7.4	71.3 ± 4.1	23.1 ± 2.1	A2A	5.6 ± 0.2	1.3 ± 0.8
β^N^/β^IVS I-1^	10	10.4 ± 2.3	31.2 ± 6.5	64.6 ± 5.1	20.7 ± 1.4	A2A	5.2 ± 0.3	1.7 ± 1.7
β^N^/β^IVS I-5^	4	10.9 ± 1.7	34.9 ± 5.4	63.0 ± 5.0	19.8 ± 1.8	A2A	5.0 ± 0.3	1.4 ± 0.4
β^N^/β^IVS II-654^	4	9.2 ± 1.1	28.0 ± 3.7	60.2 ± 3.0	19.8 ± 0.8	A2A	5.3 ± 0.3	1.8 ± 2.1
β^N^/β^N^	24	13.9 ± 2.3	40.6 ± 6.3	87.0 ± 5.6	29.7 ± 2.4	A2A	2.6 ± 0.3	0

**Table 2:** Allele frequencies of five single nucleotide polymorphisms on intragenic β-globin gene and 3′ β-globin gene in different 11 β-thalassemia genotypes in Thai population. First and second allele columns indicate ancestral allele and derived allele, respectively. Bold text with superscript * indicates Fisher’s exact test revealed DAF significant difference between wild type and β-thalassemia genotypes.

Genotypes	*n* (163)	Single Nucleotide Polymorphisms (SNPs)									
		Intragenic β-Globin Gene								3′ β-Globin Gene	
		Codon 2		IVS II-16		IVS II-74		IVS II-81		*Hinf* 1	
		C Allele	T Allele	C Allele	G Allele	G Allele	T Allele	C Allele	T Allele	T Allele	A Allele
β^E^/β^thal^	9	0.7778	0.2222 *	0.7778	0.2222	0.1667	0.8333	1.0000	0.0000	0.5000	0.5000
β^E^/β^-28^	14	0.4643	0.5357	0.4643	0.5357	0.0000	1.0000	1.0000	0.0000	0.4643	0.5357
β^E^/β^E^	31	0.8387	0.1613 *	0.8387	0.1613 *	0.0000	1.0000	1.0000	0.0000	0.8710	0.1290 *
β^N^/β^41/42^	31	0.6290	0.3710	0.6290	0.3710	0.4355	0.5645 *	1.0000	0.0000	0.1774	0.8226 *
β^N^/β^17^	23	0.2826	0.7174 *	0.2609	0.7391 *	0.0217	0.9783	0.9783	0.0217	0.1739	0.8261 *
β^N^/β^19^	4	1.0000	0.0000 *	1.0000	0.0000 *	0.0000	1.0000	1.0000	0.0000	1.0000	0.0000 *
β^N^/β^-28^	9	0.3333	0.6667	0.3333	0.6667	0.1111	0.8889	1.0000	0.0000	0.2222	0.7778
β^N^/β^IVS I-1^	10	0.7000	0.3000	0.7000	0.3000	0.3000	0.7000*	1.0000	0.0000	0.1500	0.8500 *
β^N^/β^IVS I-5^	4	0.3750	0.6250	0.3750	0.6250	0.2500	0.7500	1.0000	0.0000	0.2500	0.7500
β^N^/β^IVS II-654^	4	0.8750	0.1250 *	0.8750	0.1250 *	0.6250	0.3750 *	1.0000	0.0000	0.2500	0.7500
β^N^/β^N^	24	0.5000	0.5000	0.5417	0.4583	0.0417	0.9583	1.0000	0.0000	0.3750	0.6250

**Table 3:** Haplotype constructed using four intragenic polymorphisms on the β-globin gene and *Hinf* I polymorphism on the 3′ β-globin gene of β-thalassemia mutations and wild type in Thai population. Values indicates number of samples and (percentage).

Haplotypes	Five Polymorphisms					Frameworks	Wild Type	β-Thalassemia Mutations in the Thai Population							
	Codon 2	IVS II-16	IVS II-74	IVS II-81	*Hinf* 1 Polymorphism			Codon 26 (Hb E)	Codon 41/42 (-TTCT)	IVS I-1 (G > C)	Codon 17 (A > T)	Codon19 (A > G)	NT-28 (A > G)	IVS I-5 (G > C)	IVS II-654 (C > T)
	C > T	C > G	G > T	C > T	T > A		*n* = 133	*n* = 85	*n* = 36	*n* = 10	*n* = 25	*n* = 5	*n* = 23	*n* = 6	*n* = 4
H1	T	G	T	C	A	3a	56 (42.11%)	9 (10.59%)	10 (27.78%)		25 (100%)		23(100%)	6 (100%)	
H2	C	C	T	C	T	2	50 (38.00%)	74 (87.06%)				5 (100%)			
H3	C	C	G	C	A	1	13 (9.77%)		26 (72.22%)	5 (50%)					4 (100%)
H4	C	C	T	C	A	2	10 (7.52%)			5 (50%)					
H5	T	C	T	C	T	-	2 (1.50%)								
H6	C	G	T	C	A	-	2 (1.50%)								
H7	T	G	T	C	T	3a		2 (2.35%)							

## Data Availability

Not applicable.
